# Mindfulness-Based Mobile Applications: Literature Review and Analysis of Current Features

**DOI:** 10.2196/mhealth.2733

**Published:** 2013-11-01

**Authors:** Inmaculada Plaza, Marcelo Marcos Piva Demarzo, Paola Herrera-Mercadal, Javier García-Campayo

**Affiliations:** ^1^EduQTech R&D&I GroupDepartment of Electronics and Communications EngineeringUniversidad Zaragoza, TeruelTeruelSpain; ^2^Mente Aberta - Brazilian Center for Mindfulness and Health PromotionDepartment of Preventive MedicineUniversidade Federal de Sao Paulo - UNIFESPSao PauloBrazil; ^3^Chair of Innovation of Health TechnologiesUniversidad de ZaragozaZaragozaSpain; ^4^Instituto Aragonés de Ciencias de la SaludDepartment of PsychiatryUniversidad de ZaragozaZaragozaSpain

**Keywords:** mobile health, mHealth, mindfulness, social networks, personalized education, health informatics, evidence-based medicine

## Abstract

**Background:**

Interest in mindfulness has increased exponentially, particularly in the fields of psychology and medicine. The trait or state of mindfulness is significantly related to several indicators of psychological health, and mindfulness-based therapies are effective at preventing and treating many chronic diseases. Interest in mobile applications for health promotion and disease self-management is also growing. Despite the explosion of interest, research on both the design and potential uses of mindfulness-based mobile applications (MBMAs) is scarce.

**Objective:**

Our main objective was to study the features and functionalities of current MBMAs and compare them to current evidence-based literature in the health and clinical setting.

**Methods:**

We searched online vendor markets, scientific journal databases, and grey literature related to MBMAs. We included mobile applications that featured a mindfulness-based component related to training or daily practice of mindfulness techniques. We excluded opinion-based articles from the literature.

**Results:**

The literature search resulted in 11 eligible matches, two of which completely met our selection criteria–a pilot study designed to evaluate the feasibility of a MBMA to train the practice of “walking meditation,” and an exploratory study of an application consisting of mood reporting scales and mindfulness-based mobile therapies. The online market search eventually analyzed 50 available MBMAs. Of these, 8% (4/50) did not work, thus we only gathered information about language, downloads, or prices. The most common operating system was Android. Of the analyzed apps, 30% (15/50) have both a free and paid version. MBMAs were devoted to daily meditation practice (27/46, 59%), mindfulness training (6/46, 13%), assessments or tests (5/46, 11%), attention focus (4/46, 9%), and mixed objectives (4/46, 9%). We found 108 different resources, of which the most used were reminders, alarms, or bells (21/108, 19.4%), statistics tools (17/108, 15.7%), audio tracks (15/108, 13.9%), and educational texts (11/108, 10.2%). Daily, weekly, monthly statistics, or reports were provided by 37% (17/46) of the apps. 28% (13/46) of them permitted access to a social network. No information about sensors was available. The analyzed applications seemed not to use any external sensor. English was the only language of 78% (39/50) of the apps, and only 8% (4/50) provided information in Spanish. 20% (9/46) of the apps have interfaces that are difficult to use. No specific apps exist for professionals or, at least, for both profiles (users and professionals). We did not find any evaluations of health outcomes resulting from the use of MBMAs.

**Conclusions:**

While a wide selection of MBMAs seem to be available to interested people, this study still shows an almost complete lack of evidence supporting the usefulness of those applications. We found no randomized clinical trials evaluating the impact of these applications on mindfulness training or health indicators, and the potential for mobile mindfulness applications remains largely unexplored.

## Introduction

### Mindfulness-Based Therapies

Mindfulness techniques have emerged in the Western world in the fields of health and education as the application of ancient meditative practices from Buddhist tradition. It is from this tradition that they draw inspiration and take their basic technical features. Since its introduction, interest in mindfulness has increased exponentially particularly over the last two decades in the psychology and medicine fields [1]. Several types of approaches have been tested from secular (mindfulness-based therapies-MBTs) to Eastern meditative traditions (such as Zen and Vipassana), and scientific evidence of their effectiveness is rapidly accumulating [2,3].

The psychological trait or state of mindfulness refers to an awareness that emerges by way of paying attention intentionally and nonjudgementally, in the present moment, to the unfolding of the moment-by-moment experience [4,5]. Mindfulness is a skill that can be obtained using several training techniques, and group and individual interventions have been designed for this purpose [6]. Generally, two main complementary approaches have been used for mindfulness training (1) exercises in focused attention, and (2) open monitoring of experiences in the present moment.

Mindfulness is significantly related to several indicators of physical and psychological health such as improved immune and autonomic nervous systems, higher levels of positive affect, life satisfaction, vitality, and adaptive emotional regulation, and it has been linked to lower levels of negative affect and psychopathological symptoms [2,3,7]. Furthermore, MBTs have demonstrated effectiveness in treating many disorders, including chronic pain conditions [8-10].

The mechanisms underlying the effects of mindfulness training on health are diverse and include improvements in attention control, coping and management of life stressors, descriptions of inner experiences, thoughts and emotional awareness and regulation, and changes in the concept of the self or body awareness [11]. One of the main limitations of MBTs is the need for regular practice. Psycho-technology mobile apps have demonstrated effectiveness as a complementary tool in many psychotherapies [12], and they would be expected to be useful in MBTs as well.

### Information and Communication Technology

Information and communication technology (ICT) concerns the elements and techniques related to manipulating and transmitting information, specifically, computers, the Internet, and telecommunications. This concept is dynamic and shows rapid growth and evolution. Thus, new related paradigms are appearing, for instance, the “Internet of Things” (IoT). According to Atzori [13], the basic idea of IoT is the pervasive presence around us of a variety of things or objects—such as tags, sensors, actuators, and mobile phones—that are able to interact with one another and cooperate with their neighbors to achieve common goals. The main strength of the IoT is the large impact it will have on several aspects of everyday life including the behavior of potential users.

In this context, “smart devices” play an important role. They can perform intelligent operations and are capable of communicating to jointly deliver a service to the user [14]. Primary among these devices are “mobile devices.” They are portable, allow access to information and data anywhere, and can be carried and used during their transport. Presently, this concept includes a very large number of devices-smartphones, PDAs, MP3 players, and laptops. Most current mobile devices contain wireless communication capabilities. The common characteristics of mobile devices are their small size, portability, processing capability, network connection, and limited memory. Specifically, smartphones and small tablets allow access to a large number of apps (mobile-based software). Additionally, they can be incorporated into daily activities in a nonintrusive way.

**Figure 1:**
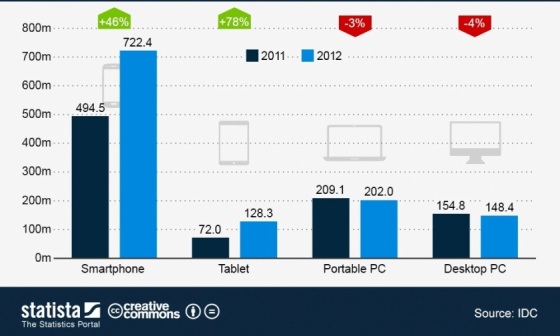
The global tablet market.

The use of mobile devices is increasing continuously. In 2012, global smartphone shipments grew 46% to 722 million units, (ie, smartphone shipments have more than tripled since 2009 when 174 million units were shipped). The tablet market also did very well in the past year. Total shipments reached 128 million units, which was a 78% increase over 2011. Conversely, the personal computer (PC) industry continued to struggle in 2012. Shipments of laptop and desktop PCs declined 3% and 4%, respectively, as consumers switched to mobile connected devices (Figure 1 shows the growing tablet market for 2011 and 2012) [15,16].

According to Milosevic et al [17], users of today’s ever-increasing number of mobile phones expect to have their favorite desktop apps on smartphones. In addition, a number of new apps are taking advantage of the specific features and sensors on smartphones. This tendency also has been observed in healthcare. A study of the Healthx Team concluded that the most recent growth in mobile apps usage has not proliferated at the expense of browsing the traditional Web; people are simply using mobile apps more [18]. Medical/health care is the third-fastest-growing app category for both the iPhone and Google Inc.’s Android phones based on information from Float Mobile Learning [19].

Currently, it is possible to search the App Store and find 21,227 apps using the criteria “Healthcare & Fitness”, 17,148 using the criteria “Medical”, or 65,128 using the criteria “Lifestyle” [20]. To design their apps, developers can choose among different platforms. The most popular are Google Android, Windows Phone, Apple iOS, BlackBerry, and Nokia’s Symbian; reference [21] gives a complete comparison of the first four.

The usability and ubiquity of mobile devices have resulted in great interest in the development of features for healthcare apps. Research has shown that disease management and health education are areas with broad potential for apps in which mobile devices could enhance the quality of life for people in general and for those living with chronic illnesses in particular [12,22]. For example, well-designed mobile apps with decision support features such as personalized education have demonstrated potential to enhance self-management outcomes in diabetes patients [22].

Healthy patients or professionals interested in MBTs or their practice may benefit from mindfulness-based mobile apps (MBMAs). The potential use of these apps could include online and offline training, practice for daily adherence and maintenance, mindfulness-based group management (for professionals), and information exchange through social forums [12].

Thus, the objective of this study is to present an in-depth analysis of the functionalities and features of current MBMAs in the most-used mobile platforms and to compare them to the current evidence-based literature regarding health and clinical settings. Comparing current with potential features and with scientific-based recommendations from the literature, we discuss a future agenda to improve the usability and utility of MBMAs.

## Methods

### Review of MBMAs

Similar to the study of Chomutare et al [22], our goal was to review MBMAs both in the scientific literature and in the most-used commercial markets and platforms. We independently searched two types of sources: (1) online journal databases, and (2) online markets. In the literature, we searched for apps that have a basis in research. In the online markets and grey literature, we searched for mature and emerging apps, new trends, and novel functionalities and features.

The main inclusion criterion for mobile apps was that they include an explicit mindfulness component. We considered the “mindfulness component” to be either secular mindfulness-based interventions with their related main techniques such as mindfulness of breathing, body scanning, walking meditation, mindful movements, and compassion-based practices or traditional mindfulness practices, such as Zen and Vipassana meditations. Regarding the MBTs, we considered all possibilities including mindfulness-based stress reduction (MBSR), mindfulness-based cognitive therapy (MBCT), acceptance and commitment therapy (ACT), and dialectal behavioral therapy (DBT).

### Review of the Literature

#### Selection Criteria

The only literatures included in this study were original research papers that addressed any mobile app with an explicit mindfulness component. We aimed to include all types of study participants including the general population, patients, and professionals. This broad inclusion criterion was settled upon after a preliminary search identified only a limited number of potential articles for inclusion. We excluded opinion-based articles and those in languages other than English, Spanish, or Portuguese.

#### Search Strategy

We searched online journal databases, indexes, and reference lists using the search terms “mindful”, “meditation”, “mobile”, “PDA”, “cell”, “phone”, and “application” from the study’s inception to April 30, 2013. We constructed a general search string with logical operators using both the conjunction “AND” and the disjunction “OR” (mindful OR meditation OR acceptance-based) AND (mobile OR PDA OR cell OR phone OR application). We adapted the search string to each database, and the search was performed on the available metadata–that is, title, abstract, and keywords.

We targeted both original research papers and review articles indexed by Medline, ScienceDirect, Embase, Psychinfo, the Association for Computing Machinery (ACM) Digital Library, the Institute of Electrical and Electronics Engineers (IEEE) Xplore Digital Library, Google Scholar and the Digital Bibliography and Library Project (DBLP) Computer Science Bibliography, and ACM Computer-human interaction (CHI) proceedings. These databases reflect the multidisciplinary nature of the research, which involves the medical, psychological, and computer science fields. We screened the reference lists of all review articles that we identified to crosscheck for missing articles of interest.

The duplicate entries from multiple databases were removed. Only original research papers were eligible for inclusion after reviewing the metadata. Any potential MBMAs not clearly reported in the metadata were explored, with assessment made of the full article. Two authors (MD and PHM) independently assessed all potentially eligible reports that were identified by the search strategy. In the case of disagreements, the matter was resolved through discussion with a third author (JGC). After the metadata screening, the eligible reports were more thoroughly analyzed by two independent reviewers (MD and PHM) who examined their full text in detail before they could finally be included. Two authors (MD and PHM) extracted the data from an included report using a predefined data extraction sheet. They also screened the reference list of all eligible reports to crosscheck for missing articles of interest. Any persistent disagreement was referred to a third author (JGC) for discussion and resolution.

#### Data Extraction and Coding

Two authors (MD and PHM) extracted data from each included report using a predefined data extraction sheet. Any persistent disagreements were referred to a third author (JGC) for discussion and resolution. All studies were coded for country and year of publication, type of study and participants, intervention characteristics, MBMA functionalities and features (as detailed below), explicit mindfulness component of the intervention, outcomes in terms of health and app usability evaluation, summary of main results, and future directions (if indicated by the authors).

#### Evaluation and Assessment of App Features and Functionalities

##### User Evaluation

We found that the evaluation of the apps is better done by the users themselves in the form of a trial or, at least, a survey. We contacted several of the most important meditation associations in Spain (including the Soto Zen Spanish Association, Kagyu and the Gelug Tibetan Buddhist Association, and Mindfulness and Health Association) and found that no one in these groups made regular use of such apps. For this reason, we ruled out the possibility of a survey and decided to revise the characteristics of the present apps. On this basis, we are developing a catalogue of possible utilities of meditation apps and, in a future research that is now in preparation, we will assess the opinion of “professional” or long-term meditators on these utilities.

Thus, we aimed to analyze the following potential MBMA features and functionalities, where available.

##### App Technology and Design

ICT features included:

1. Smartphone features and operating system
2. Text messaging or short message service (SMS); reminders or similar
3. Camera and other device for collecting and monitoring data
4. Communication tools to other mobile phone features and existing apps
5. Programming interfaces suitable for users
6. Automated sensing through sensing devices
7. Web interface for connectivity and data exchanging, and
8. Other available technology features.

Design and characteristics of functionalities included:

1. Type of mindfulness practices or interventions provided (self- and/or supervised-training; content and characteristics)
2. Tracking and monitoring personal mindfulness practice and health information (through journaling of mindfulness practicing and health-related behaviors and measures; text messaging; automated sensing and recording; online scale and questionnaires; charters and statistics; automated feedback, data export and communication with other devices or apps, synchronization with personal health record systems or patient portals)
3. Online or remote accessing to trainers or health professionals for personalized monitoring and coaching
4. Leveraging social influence (by facilitating peer-to-peer support, influence and/or modelling, integration of social media functions)
5. Increasing the accessibility to mindfulness and related health information (by informational general and/or tailored messages, reminders–content and frequency pattern, glanceable displays)
6. Utilizing any kind of entertainment as an educational tool or approach (messages with fun content, mobile phone-based video games to support mindfulness practice or related healthy behaviors)
7. Declaring “best practice” principles [23], and
8. Other available functionality.

These features and functionalities were chosen based on the relevant literature [12,22-24], mainly on the study of Klasnja and Pratt [24], and from a brainstorming and consensus process among all of the authors with two of them (MD and JGC) being experts in and teachers of MBTs. We created this list with multiple features and functionalities that we believe has the potential to enhance future MBMAs.

### Analysis of the Current Apps Available in Online Vendor Markets

#### Selection Criteria

##### Platforms

**Figure 2:**
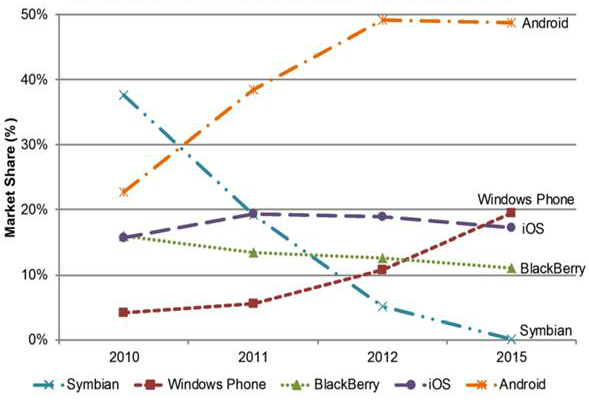
Worldwide smartphone market share forecast for 2010-2015 based on data from Gartner.

**Figure 3:**
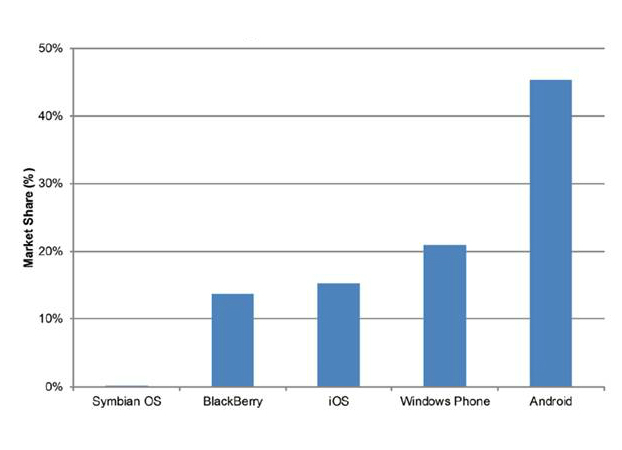
Worldwide smartphone market share forecast for 2015 based on data from IDC.

In the Introduction five operating systems were mentioned-Google Android, Windows Phone, Apple iOS, BlackBerry, and Nokia Symbian. Surveys using the latest statistics predict that Apple iOS will remain the second biggest platform worldwide, after Google Android [25,26] (Figure 2 shows this data; Figure 3 shows further data), until 2015, when Windows Phone will surpass it.

**Figure 4:**
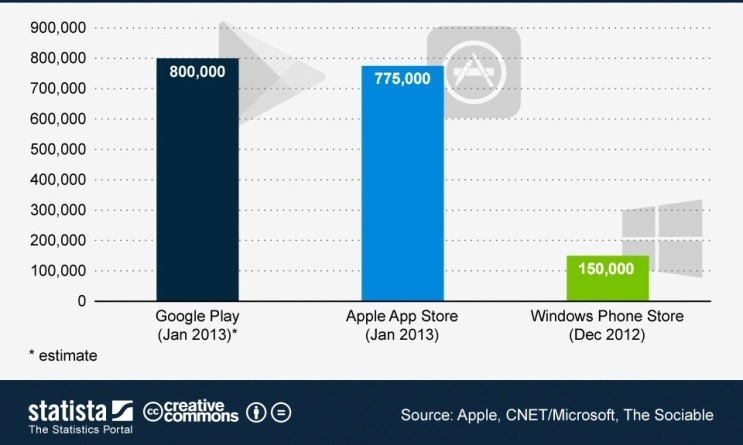
Number of apps in Google Play, Apple’s App Store, and Windows Phone Store.

In January 2013, the number of Google Android apps overtook those of Apple iOS-800,000 apps are now available on Google Play. In October 2012, Google announced that the total number of apps in its store numbered 700,000. According to a recent press release, 775,000 apps are available in Apple’s App Store. Currently, the Windows Phone Store offers 150,000 apps [27] (Figure 4 shows Google apps, Apple apps, and Windows Phone Store apps) [27].

Another important factor is the price of the smartphones that use each operating system. In terms of different models and brands, the Apple Store [28] offers the following models to users (as of March 2, 2013)-iPhone5 from €669 (iOS), iPhone4S from €569 (iOS), and iPhone4 from €389 (iOS).

At the website of the company Phone House, several mobile phones are compared and sold. Following is a sampling of several examples from different brands [29] (as of March 2, 2013)-Android (LG Optimus L3 Black €99; Samsung Galaxy Mini €119; Motorola Defy Mini Black €129; Sony Xperia Tipo Black €149; HTC Desire C 179 €179; Samsung Galaxy S III €479), Other operating systems (Nokia C2-02 Black runs on Symbian €75; BlackBerry Curve 9220 Black runs on Blackberry €179; Nokia Lumia 620 runs on Microsoft Windows Phone 8 €249), and iOS (Apple iPhone5 16Gb White €660).

In conclusion, Google Android should be considered the biggest platform worldwide for the next several years. In addition, mobile phones that use Android are less expensive than those using iOS, which contributes to that operating system’s continuous growth. Thus, this study focused on apps for Google Android smartphones. Data were obtained from the Google Play website [30] and from the selected apps.

##### Users

No restriction was applied regarding the types of users of the apps, and we aimed to include any populations, including the general population, patients, and professionals.

##### Free Apps and Paid Apps

Both free and paid apps were considered, (eg, the Mindfulness free Android and Mindfulness paid Android apps).

##### Possible Interventions or Use Cases

All possible intervention or use cases were considered including when the user is walking, sitting, or running, and at the gym, at home, or in the office.

#### Search Strategy and Data Extraction

According to the “Selection Criteria,” this study focused on apps for Google Android smartphones. Thus, data were obtained from the Google Play website. The Google Play Store classifies apps according to two main criteria: (1) “popularity” and (2) “relevance.” These criteria are based on algorithms that consider items such as the number of download, the number of uninstalled apps, and reviews from users. In this study, the “relevance” criterion was used to filter the most significant apps in two categories: (1) free and (2) paid. Finally, the research was completed in each category using the “popularity” criterion.

#### Evaluation and Assessment

As previously explained (subsection “Review of the Literature”), we contacted several of the most important meditation associations in Spain and found that no one made use of such apps on a regular basis. Thus, it was not possible to obtain the evaluation of apps either in the form of a trial or a survey. However, the Google Play Store allows users to value apps from 1 to 5. In this case, it was possible to obtain the evaluation made by users themselves based on the public Google Play results.

#### Features and Functionalities

We aimed to analyze the following potential MBMA features and functionalities for available online apps, where available: (1) number of apps, (2) number of downloads and prices, and (3) characteristics. Between characteristics: (4) main function, (5) possible interventions or use cases, (6) resources, (7) social networks, (8) contents, (9) special features, (10) outcomes: statistics and reports, (11) language, and (12) usability. Finally, (13) global assessment was chosen.

These features and functionalities were chosen based on relevant literature [22,23], our previous analyses of the possibilities that apps offer to users, and consensus process among several members of the research group EduQTech who have experience in developing mobile phone apps [31].

## Results

### Review of the Literature

**Figure 5:**
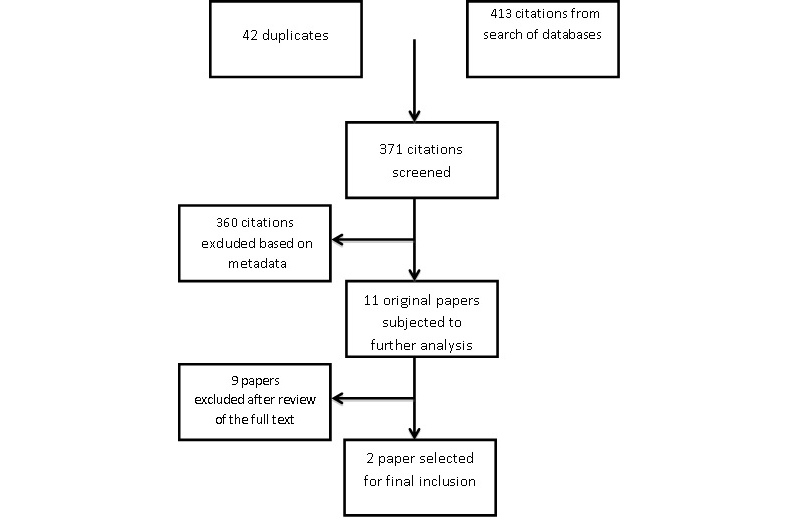
Study selection flow diagram.

We retrieved 413 citations from our database search (42 were duplicates) and excluded 360 of them based on metadata only, which left 11 eligible original papers for further analysis. We did not identify any relevant systematic review article or international guidelines related to MBMAs, and neither did we identify any new eligible reports by screening the reference lists of these 11 reports (Figure 5 shows the study selection flow diagram).

In the end, we included two original papers [32,33] in our study after excluding nine of the eligible reports after conducting a detailed analysis of their full text. The main reasons for exclusion were as follows:

1. The subject was not a true mobile app, but was instead the use of a mobile or smartphone to send and/or receive information or to access a remote website [34-37].
2. There was no explicit mindfulness component delivered [38-40].
3. The topic was mainly a computer-delivered intervention [41,42].

Multimedia Appendix 1 summarizes the general information extracted from included papers, which were: (1) a pilot study designed to evaluate the feasibility of a mobile app containing a multimedia-assisted system to support the practice of walking meditation, a type of mindfulness technique [32], and (2) an exploratory study of an app consisting of mood reporting scales and mindfulness-based mobile therapies [33]. Multimedia Appendix 2 presents a detailed description of the features and functionalities of apps, focusing on the technology and design characteristics reported in the included studies.

### Current Apps Available in Online Vendor Markets

#### Number of Apps

Table 1 shows the number of Android apps found in the Google Play App Store related to meditation, mindfulness, or similar criteria (as of February 25, 2013) [30].

To develop an in-depth analysis of all 203 available apps related to mindfulness would surpass the scope of this study. According to the “Selection Criteria” and “Search Strategy and Data Extraction” subsections, the “relevance” criterion was used to filter the 20 most significant apps in two categories: (1) free and (2) paid. Finally, 5 more were added in each category using the “popularity” criterion. Thus, 50 apps in total were analyzed in depth in this study, which is 24.6% (50/203). Multimedia Appendices 1 and 2 show the names of and relevant data for these apps. There were two free and two paid apps that did not work, thus it was not possible to develop a deep analysis of them and we only gathered information about external features (ie, language, downloads, or prices).

**Figure 6:**
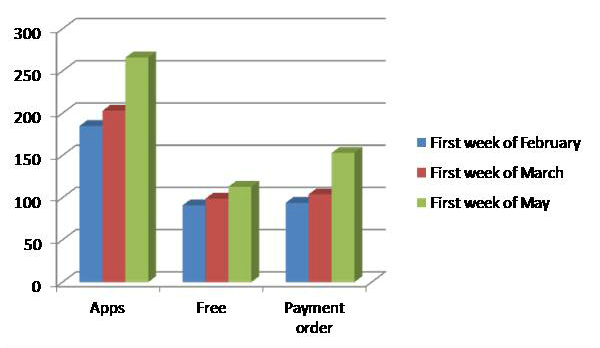
Example of the continuous evolution in the number of Android apps related to “Mindfulness.”.

Because data such as number and popularity of these apps are continuously increasing and changing, February 15, 2013 was chosen as the final date for data collection. Figure 6 shows the evolution in the number of Android apps.

#### Downloads and Prices

**Figure 7:**
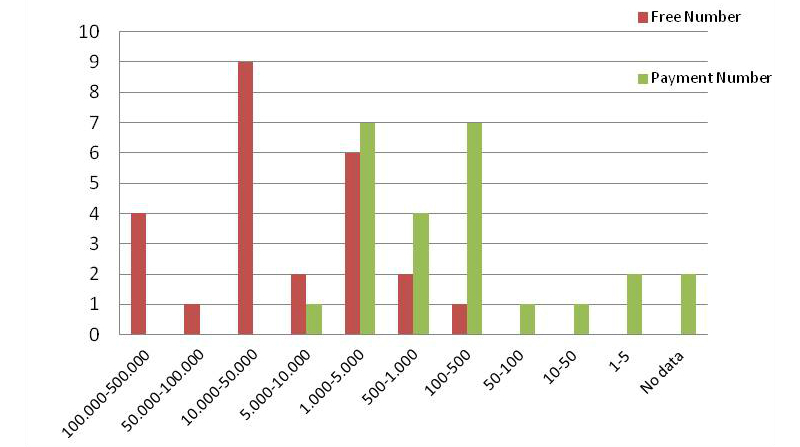
Comparison of the number of downloads among free and paid Android Mindfulness apps.

**Figure 8:**
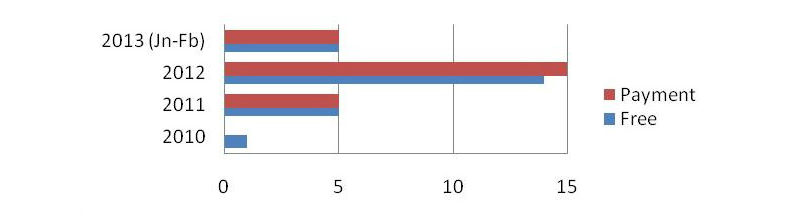
Distribution of time of most recent update.

To compare the free versus paid apps, the number of downloads was studied. Figure 7 shows it is possible to compare the download rates among the 25 apps analyzed in each case. Figure 8 shows the most recent update of these apps.

Approximately 30% (15/50) of the analyzed apps have both a free and paid version. For example, the developers of “Relax Lite: Stress Relief-Free” and “Relax: Stress & Anxiety Relief” provided interesting data about the usage of both versions [43], which are summarized in Table 2.

In this specific case, users of the paid app showed more loyalty and the number of downloads was greater than the average app. This is the only company that has provided this type of information publicly.

The prices of paid apps varied between €0.75 (in the range of 10-50 downloads) to €5.67 (in the range of 1000-5000 downloads). However, there is not a high correlation between the price and the range of downloads in general (the Pearson coefficient is only 0.34).

Conversely, approximately 52% (12/23) of the free apps offer more options after payment, and 9% (2/23) ask for donations or voluntary economic help. The most common options after payment are the following: support, additional audio, music, videos or exercises, longer meditations, subscriptions, ability to determine the duration of your session by setting a timer, removal of ads, mantras, and breath counts.

**Table 1:** Number of Google Play apps related to meditation, mindfulness, or similar criteria (as February 25, 2013).

	Apps	Free app	Paid apps
Meditation	>1000	>1000	>1000
Meditation timer	>1000	>1000	>1000
Conscience	>1000	>1000	>1000
Quality of life	>1000	>1000	>1000
Concentration	>1000	>1000	>1000
Health, healthy, health tips, health food	>1000	>1000	>1000
Breath, breathe, breathing	>1000	>1000	>1000
Health tap	>1000	>1000	325
Meditation music	>1000	>1000	46
Mindfulness	203	99	104
Meditation helper	27	15	12

**Table 2:** Information about free and paid apps from the same developer [44].

Relax Lite: stress relief–free	Relax: stress and anxiety relief
Has been downloaded over 150,000 times worldwide.	Has been downloaded over 500,000 times worldwide.
Over 25% of our users still use our apps regularly after 1 year.	Over 50% of our paid app users still use our apps regularly after 1 year.
Most apps are used by less than 5% of users after 20 days.	Most apps are used by less than 5% of users after 20 days.

#### Characteristics

##### Main Function

The term “mindfulness” or meditation was not well clarified in most of the apps. The analyzed apps had several primary functions.

Among the free apps–61% (14/23) are devoted to meditation, 13% (3/23) concern training, 9% (2/23) concern tests/questionnaires to know, for instance, the level of stress or types of stress users are experiencing, 9% (2/23) try to help focus the user’s attention, and 9% (2/23) have another main objective.

Among the paid apps–56% (13/23) are devoted to meditation, 13% (3/23) concern tests/questionnaires, 13% (3/23) concern training, 9% (2/23) are related to eating, and 9% (2/23) have another main objective.

In summary, only 56%-61% (13/23-14/23) of the apps are devoted to meditation, the core technique to develop mindfulness. The remaining apps would be best characterized with other words.

##### Possible Interventions or Use Cases

A small number of apps enabled the user to choose from among several use cases–moving, sitting, or body scan, travelling, walking, gym, or home.

All the apps are devoted to practitioners. They are not specific apps for professionals or, at least, with functionalities useful for each of the profiles.

##### Resources

**Figure 9:**
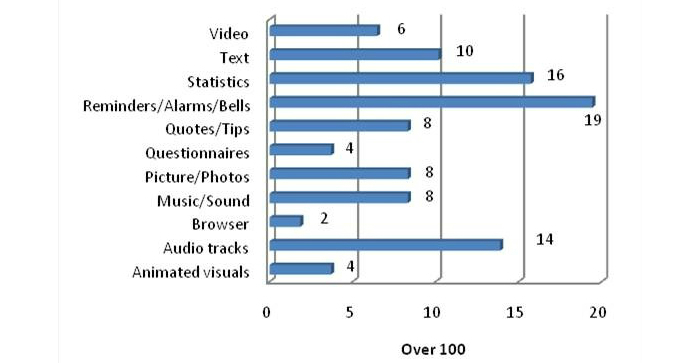
Percentages of apps that utilized different resources.

Although the design of the apps was very different, most of them used the same resources-reminders (bells, alarms, or similar), audio tracks, and text. The item “Statistics” will be explained in the statistics section. “Quotes” or “everyday tips,” “pictures or photos,” and “music or relaxing sounds” are used in a similar number of apps. Figure 9 shows the percentages of apps that utilized these types of resources. It is important to note that the video resources in several apps are links to YouTube [44].

##### Social Networks

Approximately 28% (13/46) of the apps interact with social networks. Facebook [45] and Twitter [46] are the most common according to the extracted data (see Tables 3 and 4). In addition, most of the developers provided a website.

##### Contents

In addition to the use of previously described resources, Android apps offer additional types of resources, for instance, articles, book information, events, links, personal journals, tutorials, notices, or evaluations.

##### Special Features

Several apps contained special features. Examples include breath detection, holographic theming (ie, the background and visual aspect can be changed), dieting, single (headphones) or multiple users, skill lists, and review questions about the user’s feelings after the audio and “difficulty” levels, (eg, beginner, intermediate, or advanced). However, assessments of psychological and biological variables related to mindfulness or analyses of body statics and kinetics with social interaction were not included in any app studied. The possibility of teaching or practicing for large groups via the Internet was also absent.

##### Outcomes: Statistics and Reports

Approximately 37% (17/46) of the apps present some type of statistics or reports. Usually, these reports are graphic resources or text. In Table 5, we list the graphic resources and examples of represented measurements.

The temporality is nearly always the same: daily, weekly, and monthly as well as the special case of fully customizable.

##### Language

English is the language for 41 apps. There are 11 apps that allow the use of or are developed in other languages. There are 3 apps that use Spanish, and only one app provides several videos in this language.

##### Usability

Thirteen percent (6/46) of the apps lacked an exit button and readability of the text was limited in 6% (3/46) because of the background color (4%, 2/46) or letter format (2%, 1/46).

**Table 3:** Use of Facebook.

Creation year	Followers	Social networks used together with Facebook
1996	201,290	Twitter and Pinterest
2008	643	Twitter
2009	12,742	Twitter
2010	9	Twitter (more used-768 followers)
2010	252	Twitter
2010	453	LinkedIn
2010	523	-
2011	1010	Twitter
2013	105	Google+Community and Google+Page

**Table 4:** Use of Twitter.

Tweets	Followers	Social networks used together with Twitter
1874	4137	Facebook
3524	768	Facebook
140	100	Facebook
603	936	Facebook
740	581	Facebook
264	(^a^)	(^a^)
7795	625,772	Facebook
41	28	The website does not work

^a^Last use: 2011. The access is directly through the app. For mindfulness, 45 Tweets were made in 3 days; for meditation, 14 Tweets were made in 1 hour; for relaxation, 15 Tweets were made in 1 hour; and for mindful, 15 tweets were made in 10 hours.

**Table 5:** Resources and measurements in statistical reports.

Graphic resources	Measurements
Number	Number of meditations/sessions completed
Bar chart	Time of meditation
Symbols + legend	Time versus test date/goal
Chart type and chart data	Number of consecutive days with at least one recorded session
Comments	Stress level over time or stress score out of 100 points + comments
List of choices	Time spent at each level
Use of Google	Diet: date/time/meal/snack type
Analytics	Record of the ratings: correlation analysis, performance satisfaction by intensity level, performance satisfaction by focus level, and determination of whether the intensity level has positive or negative impact by comparing with the focus.

#### Global Assessment

##### Valuing Mobile Apps

To study the global assessment by users of the analyzed apps, we were able to employ the data that Google Play Store offers online. As explained in the Methods Section, users can value mobile apps from 1 to 5.

Multimedia Appendices 3 and 4 show the global score and the number of votes for each analyzed app. In total 10,984 assessments were found, 10,309 corresponding to the free versions and 675 to the paid versions of the apps.

In the free versions the scores ranged from 1.2 (lowest) to 5.0 (highest). There were 10,309 users that had assessed the apps, and the average was 4.6. In the paid versions the scores ranged from 1.7 (lowest) to 5.0 (highest). There were 675 users that had assessed the apps and the average was 4.0.

Users experienced higher satisfaction with the free apps.

##### A Case

The most relevant free app has a score of 4.5 (88 voters). The app allows users to utilize its Facebook Page (created in 2013 and had 105 followers in January 2013), Google+Community and Google+Page. The developers also provide a “Journal” where users can score their “Conscious level” (from 1 to 7), add comments about the practice (challenge), and know at any time the number of people using the program.

In Table 6, the number of “conscious people” is shown together with the number of people that completed a challenge. Sunday data are not shown because of technological problems.

It is interesting to note that Thursday and Saturday are the days with lower numbers of users, but higher percentages of completed challenges. On the rest of the days, the percentage of users who completed the challenge was approximately 37.22% (590/1585). In other words, only one-third of the followers completed the challenge.

**Table 6:** Average of the maximum number of users and the percentage who completed the daily challenge.

	Average number of simultaneous users	% of users who completed the daily challenge journal
Tuesday	434	39
Wednesday	407	38
Friday	390	33
Monday	354	39
Thursday	271	65
Saturday	228	58

## Discussion

### Usefulness of MBMAs

Mobile apps may be useful at facilitating mindfulness accessibility, training, and daily practice adherence [39], which could enhance the impact of MBTs on health indicators worldwide. While a wide selection of MBMAs seems to be available in the market, this study finds a complete lack of evidence to support the usefulness of those apps. We did not find any randomized clinical trials that evaluate the impact of mobile apps and its features and functionalities on mindfulness training or health indicators. In fact, we found only two small-sample pilot studies [32,33]. The study by Yu et al [32] was designed to evaluate the feasibility of an MBMA with a multimedia-assisted system to train and support the practice of walking meditation, a type of mindfulness technique. As that multimedia-assisted system uses a complex net of sensors, it will most likely remain useful only for mindfulness research rather than for the general population. Another study, by Morris et al [33], whose MBMA is probably more suitable to the general population, explored the feasibility of an app consisted of mood reporting scales using touch screen, and self-guided and -demanded mindfulness-based mobile therapies. Both MBMAs were developed for the Android operating system, following the tendency observed in the online vendors market.

The lack of evidence we have found is probably because both MBTs and their related mobile apps are in their early technological development phase, and because developers in the vendors market are not involved in academic or health settings. In our opinion, present apps are not aimed to usual meditators but to nonmeditators that want to become introduced to this technique, because the market is wider. Most of the features and functionalities expected and required for meditators were absent from existing MBMAs. There is ample room to explore in regard to the usability and effectiveness of mobile apps [47]. The way to do this is to identify present functionalities, as we have done in this manuscript, suggest new functionalities available with present technology, and ask meditators about their feasibility and usefulness, which is part of a new project.

We identified 203 eligible MBMAs in the online market (February 25, 2013). It is interesting to note that the number of mindfulness apps is much lower than the number of apps found using other related criteria. As expected, it is clear that the number of free downloads is several orders of magnitude higher than paid downloads. It is also worth pointing out that free versions obtain better scores than paid apps. The current time is exciting as the number of apps is increasing quickly, as shown by the data from 2011 versus the data from two months in 2013 (Figure 8). Looking at the same figure, it is also possible to predict a trend towards the development of more paid apps. In this category (paid apps) a more expensive app does not lead to a lower number of downloads (see Results section, subsection Downloads and Prices).

“Mindfulness” terms and definitions that apps use to self-classify as MBMAs were not always well clarified. Thus, nearly half of the analyzed apps presented different main functionalities and features that were not necessarily related to mindfulness/meditation. It will be necessary for developers to clarify the mindfulness concept and the main objectives of mindfulness apps to better meet the expectations of potential users, as satisfaction can be considered one of the most important parameters for usability [35]. It is interesting to note that the term “mindfulness” was absent in the metadata of the paper from Morris et al [33]; in fact, it is absent throughout the article. This means that terms and definitions for mindfulness are also an issue in the scientific literature, and future systematic reviews on this subject should perform an exhaustive search to prevent this potential bias in selection. In addition, authors should also include the term “mindfulness” or similar in the metadata whenever this is appropriate and relevant.

Some aspects of usability deserve some comments–a minor percentage (about 20%, 9/46) of the apps would benefit from improvements to the design of the interface, (eg, clear exit buttons, changes to the background color or legibility in order to facilitate reading the text, etc). As these pitfalls are subjective and rather difficult to evaluate, two authors, to increase reliability, assessed them. They are easy to improve and would make these apps friendlier.

The question of the language used bears a relation with usability. As 78% (39/50) of the apps studied were only available in English, users speaking other languages would have more difficulties with these apps. This is an important feature because mindfulness decreases conceptual language [5], and it could be more difficult to practice mindfulness while thinking in a language different from the user’s native tongue.

As regular practice is a key element in obtaining health benefits from MBTs, it is considered a very important issue among available MBMAs, as approximately 37% (17/46) of them presented some types of statistics or reports of the user’s mindfulness practice. Another key element appears to be the use of social networks as a tool to promote contact among users, to create a sense of community and to permit the exchange of mindfulness practice experiences between users. Therefore, “tracking and monitoring personal mindfulness practice and health information” and “leveraging social influence” seem to be important functionalities for the MBMA developers in the market.

Assessments of psychological and biological variables related to mindfulness or analyses of body statics and kinetics with social interaction are not included in any app studied, except the study of Morris et al [33], which evaluated users’ mood reporting. The lack of these functionalities, which are relevant for research studies and for feedback from users, might be one of the reasons for the absence of randomized clinical trials to evaluate the impact of mobile apps on mindfulness training. The improvement of health outcomes produced by apps is probably linked to empowerment and interactivity with the smartphone [47], and psychological and biological feedback could be one of the most important rewards to encourage users to continue to practice mindfulness.

Finally, no app offers a relevant functionality for instructors of mindfulness-the possibility of teaching or practicing for large groups via the Internet. It is widely accepted that the future of psychotherapy is linked to smartphones [12], so this functionality is expected to be one that generates the most demand from trainers and trainees of mindfulness.

### Limitations

A limitation of this review is that we did not include an extensive evaluation of websites related to mindfulness training in our method because that would be beyond the scope of this work. As a result, it is possible that we are missing some MBMAs not currently available in online markets or in scientific databases. Furthermore, some functionalities and features may be underreported as we did not thoroughly evaluate all of the MBMAs available in the online vendor markets.

### Existing MBMA Versus Real Needs: Future Agenda for Research

Mobile phones and apps are widely used to enhance the delivery of mental health prevention and treatment interventions [48], and they will be a key element in future research on health behavior, according to the experts [12]. These devices are ubiquitous, well accepted in society, relatively inexpensive, programmable and capable of recording media, are already owned by a large number of people, operate almost continuously, allow the input and output of data, and provide content that is more user-friendly and condensed than regular Web pages [39]. With a specific focus on apps that increase the adherence and efficacy of mindfulness practices, there are important limitations in the current apps that future research should address. Some improvements in new apps that would facilitate mindfulness training, practice, and research are the following.

Monitoring psychological variables related to mindfulness would be feasible with present technology and quite useful to meditators that wish to periodically assess their levels of mindfulness or other psychological constructs, such as acceptance, psychological flexibility, and resilience. The user’s emotional state could even be assessed, for example, with emotion recognition by voice or photographs obtained with mobile microphones and cameras [49].

Monitoring of biological variables through internal and external sensors could give useful psychophysiological data. Consumer electroencephalogram (EEG) headsets and electrocardiogram (ECG) monitors have already been developed [50,51] and could allow monitoring of brain and heart activity during formal and informal mindfulness practices. Changes in EEG and ECG can be expected as a consequence of mindfulness practice over the acute/medium/long term. Experts consider [12] that, in the future, many people will have a set of wearable wireless biosensors that will monitor not only basic vital signs (temperature, blood pressure, pulse, respiratory rate, pulse ox, and ECG), but also other variables relevant to mindfulness (hormone levels, immune system activity, and inflammation) [52].

Monitoring of physical movement and the psychological state with MBMAs associated with 3-axis accelerometers (to detect linear acceleration in all directions) could reveal whether people are practicing while standing, moving, or walking and whether they feel calm or nervous [53,54].

The analysis of body statics and kinetics could be possible in the near future. Devices for full-body gaming such as the Xbox 360 Kinect will soon be connectable to mobile devices, which would make possible studies of nonverbal behavior according to body position, posture, and motor skill learning. It may also be possible to record limb and torso movements continually to detect changes in body statics and kinetics due to mindfulness practice [12].

The analysis of social interactions could be used by trainers. Devices with Bluetooth routinely scan the identity codes of all other Bluetooth devices nearby. As most people now carry a Bluetooth-enabled mobile phone, the density of local Bluetooth devices is a good proxy for the number of people nearby [55]. This information and call log data allow the inference of social networks associated with mindfulness practice [56] and could be useful for trainers to control individual presence during group activities related to mindfulness training.

Improving adherence to practices can be done online. Achieving individual improvements through online “situational feedback” delivered by trainers, automatic SMS alerts, or a reminder is a functionality that already exists and may improve adherence to a regular mindfulness practice [24,36].

Context-specific and customized behavior activation can help app users. Context-specific alerts, SMS, reminders, tips, and educational content probably will make these functionalities more effective, and customized feedback will improve users’ skills to be more mindful during, or to practice mindfulness exercises just before well-known stressful moments or unhealthy behavior daily triggers (for example, preventing psychological overreaction to a known stressful social event) [24].

### Conclusions

While a wide selection of MBMAs is available in the online market, this study found that there is still a lack of scientific evidence to support the use and effectiveness of those apps.

### Recommendations

According to the results of this study, we provide the following recommendations for future apps.

It is necessary to clarify the concept of mindfulness and the principal aim of any mindfulness app to better meet the expectations of potential users. About 41% (19/46) of the apps that include mindfulness in the title develop some form of a mind-body approach, but not specifically mindfulness.

About 20% (9/46) of the apps have interfaces that are difficult to use, for instance, there is no clear exit button or the background color makes reading the text difficult. To solve these problems, published guidelines for app design could be developed (see, for instance, Google, Apple, and Windows) [57-59] or [17]. Smartphone apps must be intuitive to increase everyday use and generate confidence.

No specific apps exist for professionals or long-term meditators or, at least, for both profiles (users and professionals) and contain functionalities useful for each.

The functionalities of the apps available at present are limited. Assessments of psychological and biological variables related to mindfulness or analyses of body statics and kinetics with social interaction are not included in any app studied, except one. Another relevant functionality for instructors of mindfulness, the possibility of teaching or practicing for large groups via the Internet, is also absent.

It is necessary to develop apps in languages other than English (only 22%, 11/50 of the apps studied allow any other language) to make them friendlier to non-English-speaking users.

In summary, apps are expected to be an important additional tool to increase adherence in MBTs, but some improvements to the present models are advisable to improve their efficacy. The potential for mobile mindfulness apps remains largely unexplored.

## Multimedia Appendix 1

General characteristics of studies.

## Multimedia Appendix 2

Apps features and functionalities (technology and design characteristics) from the included studies.

## Multimedia Appendix 3

Mindfulness free Android apps.

## Multimedia Appendix 4

Mindfulness paid Android apps.

## Abbreviations

ACM: Association for Computing Machinery
ECG: electrocardiogram
EEG: electroencephalogram
ICT: Information and communication technology
IoT: Internet of Things
MBMAs: mindfulness-based mobile apps
MBTs: mindfulness-based therapies
PC: personal computer
SMS: short message service
