# Supplementary material for: Mindfulness-Based Mobile Applications: Literature Review and Analysis of Current Features
Source: JMIR Mhealth Uhealth. 2013 Nov 1;1(2):e24. doi: 10.2196/mhealth.2733 (PMC4114453; doi:10.2196/mhealth.2733)
Supplement: Supplementary file 1 [file mhealth_v1i2e24_app1.pdf]

Table 1. General characteristics of studies.

Country/Year	Type of study (duration/phone provided)	Subject type (number of subjects/health condition/ recruitment)	Intervention	Mindfulness component	Outcomes (health)	Outcomes (app evaluation)	Summary of results	Future Directions
Taiwan, 2012	Experiment 1: controlled study, 15 participants in each group<br><br>Experiment 2: pre-post study - four different tasks were evaluated (1) walking with no multimedia assistance; (2) walking with auditory guidance only; (3) walking with visual guidance only; and (4) walking with visual-auditory guidance	Experiment 1: 30 participants - average age was 25.2 (SD=3.71) - beginners in meditation techniques.<br><br>Experiment 2: 6 participants – mean age 26.2 (SD = 2.56) - meditators with no previous experience at synchronizing their walking rhythm with breathing during walking meditation.<br><br>Recruitment method and health status of participants not reported.	"Walking meditation" training using a mobile app with a multimedia-assisted system	"Walking meditation" (a type of meditation which aims to be as slow and conscious as possible while taking steps)	Not evaluated	Experiment 1: -to evaluate whether the multimedia-assisted mechanism is capable of enhancing a beginner's walking awareness through walking meditation (variables: stride time and incorrect footsteps)<br><br>Experiment 2: to evaluate better feedback mechanisms to learn the techniques of Breathwalk through walking meditation (variables: footstep retardation time, inhalation and exhalation retardation time, degree of shakiness, and incorrect footsteps in each task)	(1) The system effectively assisted beginners in slowing down their walking speed and decreasing incorrect footsteps; (2) the visual-auditory mechanism appears to be a better multimedia-assisted mechanism to teach walking meditation than the visual mechanism or auditory mechanism	(1) To evaluate the meditation conditions through EEG measurements, for example; (2) to develop a hands-free visual feedback mechanism through the use of, for example, a pico-projector, glasses, or lasers
USA, 2010	Exploratory study (one-month field study) mixing qualitative and quantitative methods (weekly open-ended interviews and longitudinal collection of data). An HTC 3600 mobile phone was provided to participants (phones could not be used for phone calls because of complications associated with transferring calling plans).	Out of ten initial participants (six women, ages ranged from 30 to 48 years, mean age=37/SD= 5.75), eight completed the study (two discontinued participation due to time constraints). Participants were recruited from a sample of employees who had reported significant stress levels (stress level as 3 or higher on a scale of 1 to 5, assessed by the Mayo Clinic Health Risk Assessment tool).	Use of an app consisting of mood reporting scales and mobile therapies. The mood reporting scales included the Mood Map and single-dimension mood scales (see table 2 for details). The experience sampling app pushed these scales to participants at scheduled times in the morning, evening and throughout the day. Once participants recognized their moods, they could access the 3 "mobile therapies" by touching icons on the main screen of the app	All 3 "mobiles therapies" present mindfulness-based components (1) the breathing exercise, a blue circle that expanded and contracted slowly to encourage deliberate and slower breathing, resembles with "mindfulness of breathing", a main mindfulness-based exercise; (2) the "Body Scan" resembles to the mindfulness exercise called also "body scan" that encourages progressively awareness on different	Characteristic patterns of mood change over the course of the one-month study, the diurnal cycle, and during specific stressful incidents.	(1) The app logged the time and date of all user interactions to assess use patterns. (2) Open-ended interviews were performed to evaluate how participants had used the phone app to reflect on their moods and handle stressful situations, and to identify other ways they had shaped the app to the nuances of their lives. Participants shared reactions to the app and to trends of their experience sampling data, which were shown on a laptop computer.	(1) Individuals varied considerably in the frequency of their responses: over the course of the study, the number of mood scale responses ranged from 412 to 828 with a median of 612. Most participants used the app in spurts rather than steadily. On average, participants completed 21 mood scales per day. (2) A number of participants reported changes over the course of the study in their mood patterns and coping skills, and ascribed these changes	Future systems could combine the assessment, mobile therapies, and feedback from interviews that could be built into the software and used to customize the mobile therapies. To cultivate self-awareness the system should ideally present mood trends on the phone immediately after a mood entry. The system should also invite users to investigate their mood correlates and set goals. To help with managing situation-specific stressors, feedback

			(short translations of cognitive behavioral therapy concepts adapted to the mobile phone) (1) breathing visualization, (2) a physical relaxation animation called the Body Scan, and, (3) a series of cognitive reappraisal exercises called the Mind Scan.	areas of the body; and (3) the "Mind Scan", although mainly a cognitive approach, can be associated to the cognitive defusion techniques trained during mindfulness-based exercises.			to use of the app. (3) Five case studies illustrate participants' use of the mobile phone app to increase self-awareness and to cope with stress. Similar changes were observed among other participants as they used the app to negotiate bureaucratic frustrations, work tensions and personal relationships. Participants appeared to understand the mood scales developed for this experience sampling app and responded to them in a way that was generally consistent with self-reflection in weekly interviews. Participants quickly grasped the Mood Mapping and therapeutic concepts, and applied them creatively in order to help themselves and empathize with others.	displays could illuminate the contextual triggers and help the user to develop coping strategies. The system could track which therapies were most helpful and provide similar but increasingly sophisticated strategies over time. Experimental studies to assess the potential benefits of such systems are recommended, in addition to larger field deployments to understand how such systems might be adopted in communities. Among other topics that can be examined in qualitative field studies is mood sharing, that is, how people use their Mood Map ratings or media to represent their emotional states, the clusters of people with whom they share mood data, and the contagion effects of mood in social networks.

SD=standard deviation
