# Supplementary material for: Mindfulness-Based Mobile Applications: Literature Review and Analysis of Current Features
Source: JMIR Mhealth Uhealth. 2013 Nov 1;1(2):e24. doi: 10.2196/mhealth.2733 (PMC4114453; doi:10.2196/mhealth.2733)
Supplement: Supplementary file 2 [file mhealth_v1i2e24_app2.pdf]

Table 2. Apps features and functionalities (technology and design characteristics) from the included studies.

				TECHNOLOGY								DESIGN				
Country / Year	Smartphone features and operating system	Text messaging (SMS), reminders or similar	Camera and other device for collecting and monitoring data	Communication tools to other mobile phone features and existing apps	Programming interfaces (APIs) suitable for users	Automated sensing through sensing devices	Web interface for connectivity and data exchanging	Other available technology features	Type of mindfulness practices or interventions provided (self- and/or supervised-training; content and characteristics)	Tracking and monitoring personal mindfulness practice and health information	Online or remote accessing to trainers or health professionals for personalized monitoring and coaching	Leveraging social influence (by facilitating peer-to-peer support, influence and/or modelling, integration of social media functions)	Increasing the accessibility to mindfulness and related health information (by informational general and /or tailored messages, reminders -content and frequency pattern-, glanceable displays)	Utilizing any kind of entertainment as an educational tool or approach (messages with fun content, mobile phone-based video games to support mindfulness practice or related healthy behaviors)	Declaring "best practice" principles	Other available functionality
Taiwan, 2012	HTC Desire HD running Android 2.3 with a 4.3-in LCD screen	NA	NA (see sensors)	NA	NR	(1) A pair of sensing shoes that detect the force distributions of the feet. In addition, it transfers the sensed values to the relay wirelessly.	NA	To provide multimedia guidance and measures timed reaction to it by three	Self-guided "Walking Meditation" (to improve awareness of walking conditions by watching visual feedback in	To detect a user's motion (it can detect the footstep frequency and speed - footsteps per minute - and walking method in	NA	NA	NA	NA	NR	(1) To help beginners to synchronize footsteps with breathing and to land every footstep with toes

						The sensing module is based on Atmel's high-performance, low-power, 8-bit AVR ATMega328 microcontroller and a 2.4-GHz Zigbee 1-mW chip antenna module. The module size is 2.2 cm × 3.5 cm × 0.5 cm with an overall weight of 85 g; (2) a breathing garment (includes a respiratory girth sensor (RGS), a medical instrument, and a Bluetooth module)		types of feedback mechanisms are designed to show the breathing and walking information: visual mechanism, auditory mechanism, and visual-auditory mechanism	real time)	real time), breathing conditions (measurement of the elongation of the thorax caused by breathing - breathing rate and depth, inhalation and exhalation time and their ratio can be obtained), and the remaining time of meditation						first; and (2) to allow breathing rhythm control (for example, the user can set 3 s for inhalation and 4 s for exhalation)
USA, 2010	HTC 3600 mobile phone (Android operating syste	NA	Touch screen.	NA	NA	NA	NA	NA	Self-guided mindfulness-based exercises: (1) "breathing exercise", a blue circle that expanded	General expression of emotional experience captured by touch screen scales for mood reporting: (1)	NA	NA	NA	NA	NA	NA

m not detailed).	and contracted slowly to encourage deliberate and slower breathing; (2) "Body Scan" that includes an outline of a human figure with rhetorical questions about where the user might be holding tension, and as the user clicks through the questions, that section of the body outline changed from red to blue (resemblance to the classical mindfulness-based exercise also called "body scan"); and (3) the "Mind Scan", although mainly a	Mood Map (a touch-screen translation of the circumplex model of emotion, where participants describe their mood by indicating its location on a two-dimensional space formed by the horizontal axis of “negative–positive” and the vertical axis of “high–low” energy), and (2) single-dimension mood scales for happiness, sadness, anxiety, and anger (arranged vertically on the screen with an 11-point range with all entries made via the touch screen). The

cognitive approach, can be associated to the cognitive defusion techniques trained during mindfulness-based exercises.	experience sampling app pushed these scales to participants at scheduled times in the morning, evening and throughout the day.

NA=not available
NR=not reported
