# Supplementary figures and images for: Mindfulness-Based Mobile Applications: Literature Review and Analysis of Current Features
Source: JMIR Mhealth Uhealth. 2013 Nov 1;1(2):e24. doi: 10.2196/mhealth.2733 (PMC4114453; doi:10.2196/mhealth.2733)

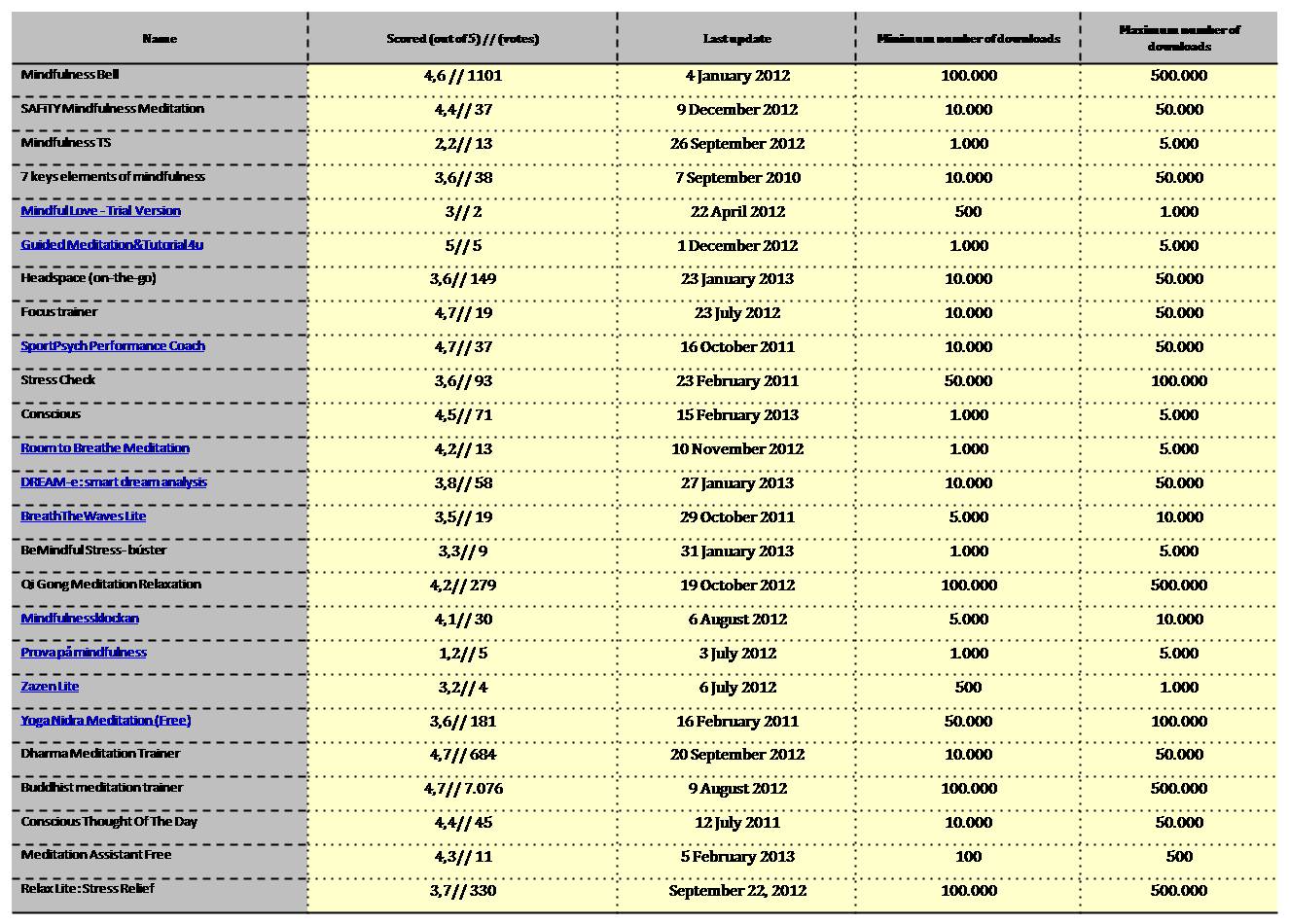

Supplement: Supplementary file 3 [file mhealth_v1i2e24_app3.jpg]

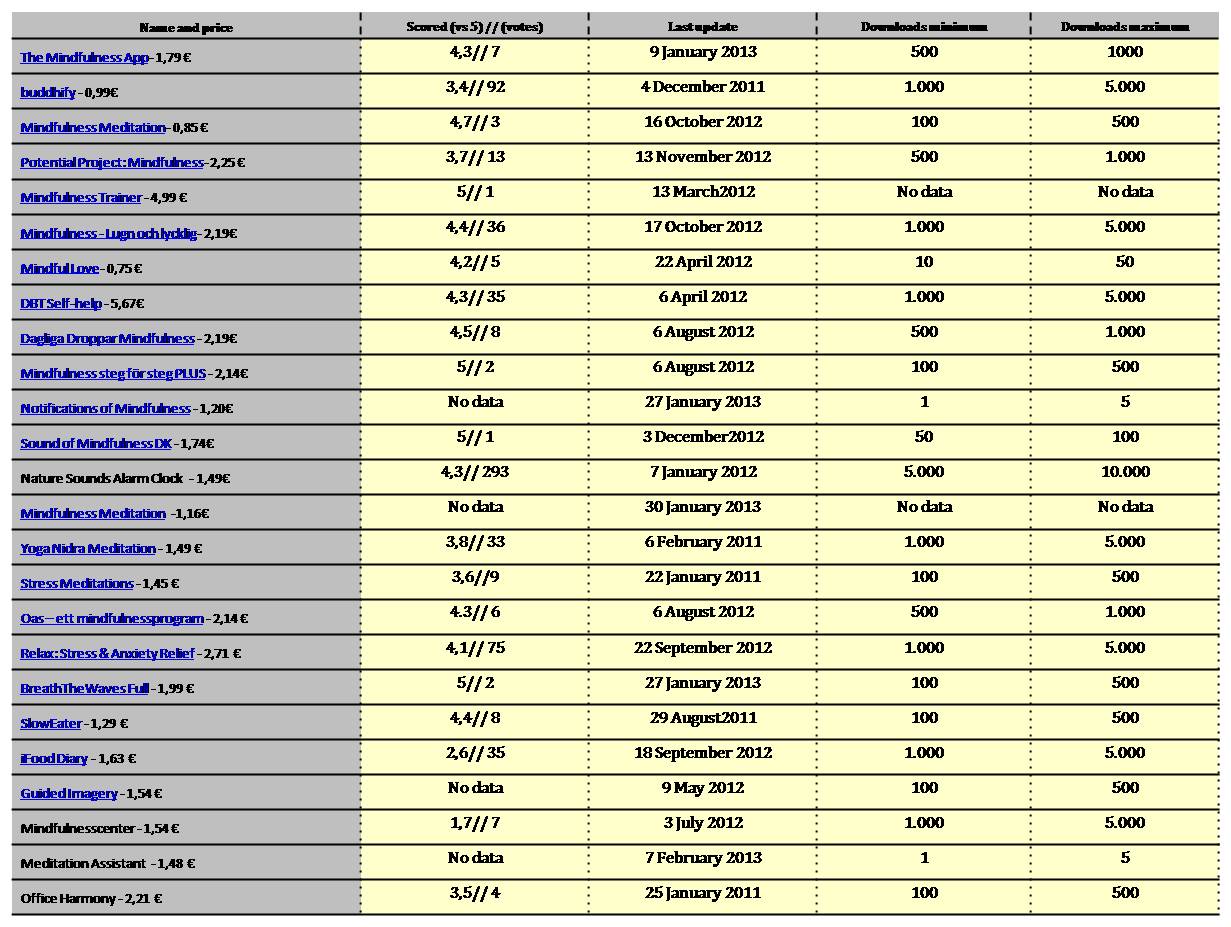

Supplement: Supplementary file 4 [file mhealth_v1i2e24_app4.jpg]
